# Integrated fever management: disease severity markers to triage children with malaria and non-malarial febrile illness

**DOI:** 10.1186/s12936-018-2488-x

**Published:** 2018-10-10

**Authors:** Chloe R. McDonald, Andrea Weckman, Melissa Richard-Greenblatt, Aleksandra Leligdowicz, Kevin C. Kain

**Affiliations:** 1 SAR Laboratories, Sandra Rotman Centre for Global Health, University Health Network-Toronto General Hospital, MaRS Centre, TMDT, 10th Floor 10-351, Toronto, ON M5G 1L7 Canada; 2 Department of Laboratory Medicine and Pathobiology, University of Toronto, Toronto, Canada; 3 Interdepartmental Division of Critical Care Medicine, University of Toronto, Toronto, Canada; 4 Tropical Disease Unit, Division of Infectious Diseases, Department of Medicine, University of Toronto, Toronto, Canada; 5 Toronto General Research Institute, Toronto General Hospital, Toronto, Canada

**Keywords:** Malaria, Disease severity, Severe malaria, Innate immunity, Inflammation, Endothelial activation

## Abstract

Febrile symptoms in children are a leading cause of health-care seeking behaviour worldwide. The majority of febrile illnesses are uncomplicated and self-limited, without the need for referral or hospital admission. However, current diagnostic tools are unable to identify which febrile children have self-limited infection and which children are at risk of progressing to life-threatening infections, such as severe malaria. This paper describes the need for a simple community-based tool that can improve the early recognition and triage of febrile children, with either malarial or non-malarial illness, at risk of critical illness. The integration of a disease severity marker into existing malaria rapid diagnostic tests (RDT) could enable detection of children at risk of severe infection in the hospital and community, irrespective of aetiology. Incorporation of a disease severity marker could inform individualized management and early triage of children at risk of life-threatening infection. A child positive for both malaria and a disease severity marker could be prioritized for urgent referral/admission and parenteral therapy. A child positive for malaria and negative for a disease severity marker could be managed conservatively, as an out-patient, with oral anti-malarial therapy. An RDT with a disease severity marker could facilitate an integrated community-based approach to fever syndromes and improve early recognition, risk stratification, and prompt treatment of severe malaria and other life-threatening infections.

## Background

### Statement of problem

Febrile syndromes account for over 1 billion episodes annually and are one of the most common reasons to seek medical care worldwide [1, 2]. A child in sub-Saharan Africa will experience a mean of 5.9 episodes of fever each year, translating into more than 660 million episodes annually across the sub-continent [2, 3]. The majority of these febrile illnesses are uncomplicated and self-limited, and only a small proportion of children progress to serious infections including severe malaria. However, there is a lack of rapid and reliable tools to identify which children have, or are progressing to, life-threatening infections. This is a major barrier to rational triage and management of fever syndromes and results in increased mortality in those with severe infections and the misallocation of scarce health resources due to inappropriate referral, admission and/or antimicrobial treatment of patients with self-limited infections, resulting in harm, increased health care costs and antimicrobial resistance.

### Proposed solution

This paper advocates for the development of rapid and simple tools to enhance the early recognition and triage of severe malaria and other life-threatening infections in the community setting. This approach would permit integrated community-based management of “all cause” fever syndromes. By avoiding unnecessary referral of self-limited and uncomplicated infections, it would decompress health care facilities, while focusing health resources on those at risk of severe infection that are most likely to benefit from referral, admission and supportive care. This strategy could save lives and health dollars.

### Severity markers to triage fever syndromes

#### Severe malaria as a model-challenges and opportunities

**Fig. 1:**
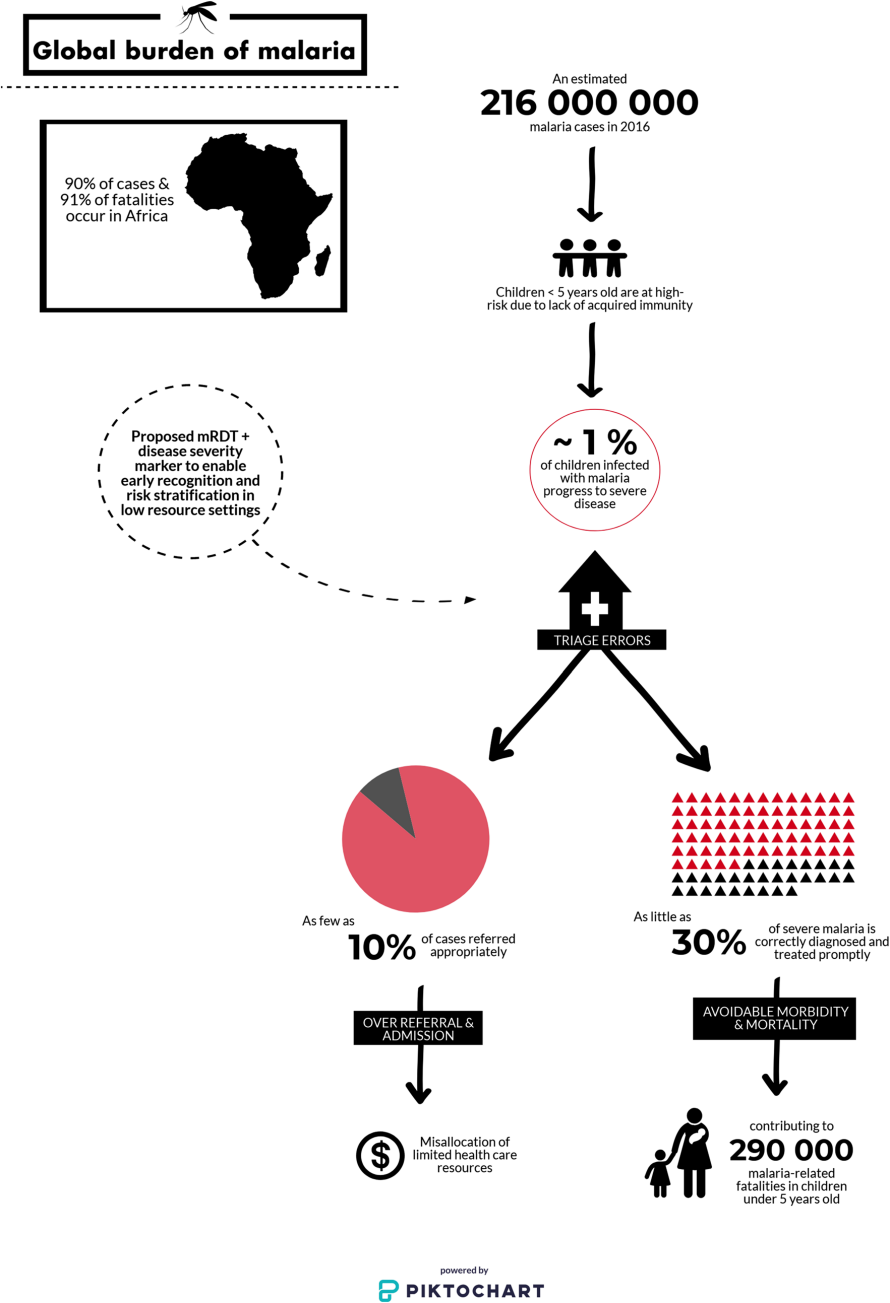
Overview of the global burden of malaria in children under five. There were an estimated 216 million cases of malaria infection in 2016. The majority of malaria cases and fatalities occur in sub-Saharan Africa. An estimated ≤ 1% of all malaria infections present with or progress to severe disease, which is associated with an increased risk of morbidity and mortality. Current barriers to improved management and outcome for paediatric febrile illnesses include the lack of simple and rapid tools to enhance triage and referral of severe malaria and other life-threatening infections [7], Licence: CC BY-NC-SA 3.0 IGO

Malaria remains a major contributor to childhood death and disability [4]. In 2016 there were over 216 million reported cases of malaria infection resulting in 290,000 deaths in children under the age of five (Fig. 1). Up to one quarter of all children who survive severe malaria experience long-term neurological sequelae including impaired learning, epilepsy, and increased risk of behavioural disorders [4–6].

As a result of current malaria control and elimination efforts, malaria prevalence is changing and the proportion of fevers due to malaria is decreasing in many regions of Africa (ranging from ≤ 10 to > 70% [7]). The roll out of malaria rapid diagnostic tests (RDT) has enhanced the management of uncomplicated malaria, but has also created new challenges; in particular how best to manage the large number of children with non-malarial fevers. This barrier has resulted in an escalation of inappropriate antibiotic use. For example, in an analysis of observational and randomized studies that included over 500,000 febrile participants, antibiotics were prescribed to 69% of patients who were RDT negative, with lower anti-malarial use being replaced with increased antibiotic use [8, 9].

An integrated approach is required to provide rational management of both malaria positive and malaria-negative cases and enhance the early recognition and triage of severe infections.

#### Current malaria RDTs cannot diagnose severe disease

The early recognition and treatment of children with severe malaria can improve survival but, like sepsis, the initial presentation may be subtle and non-specific [2, 10–13]. Malaria diagnosis across many hospital and community-based settings relies on RDTs that detect circulating parasite antigens. While pathogen-based tests have transformed diagnosis, they do not advise management beyond the presence or absence of infection. Specifically they do not inform critical management decisions regarding patients who have, or are progressing to severe disease, and consequently need urgent referral/admission and parenteral therapy. In an era of “patient first medicine”, a sole focus on the pathogen is not a small problem. For example, in a large survey of severe malaria management in 103 health units in Uganda, referral practices to formal health care centers were reported to be appropriate in less than 10% of cases, while less than 30% of those with severe malaria were diagnosed and treated promptly [14]. Similar problems may exist elsewhere in malaria-endemic areas [12, 15, 16]. As with other life-threatening infections, delays in the recognition and treatment of severe malaria result in increased mortality and long-term morbidity in survivors [10, 11, 17], whereas over-referral and admission of uncomplicated cases misallocates limited health resources and causes harm [10, 14].

#### Endothelial and immune activation markers can identify patients with severe malaria

Clinical evaluation of infection-related disease severity remains imprecise in paediatric and adult populations in both low and high resource settings, indicating a need for more accurate tools [13, 14]. The outcome of any infection depends on a complex interplay between the pathogen and host. Host response is a critical determinant of the onset and outcome of severe infections and several lines of evidence indicate that life-threatening infections share common pathways of host response leading to end-organ injury [18–29]. Of these shared pathways, endothelial and immune activation have emerged as key contributors to the pathogenesis of severe and fatal infections [24–30]. Endothelial and immune activation precedes the loss of endothelial integrity, microvascular leak, multi-organ dysfunction and death [28, 30–32]. Markers of these pathways (e.g. Angiopoietin/Tie2) have been shown to be independent and quantitative markers of disease severity and prognosis, not only in in *Plasmodium falciparum* malaria, but also in *Plasmodium vivax* and *Plasmodium knowlesi*, as well as sepsis and other infections [21, 22, 26, 30–39]. Moreover, unlike C-reactive protein (CRP) and procalcitonin (PCT), these markers are actual pathway mediators and therefore represent “druggable” targets to improve the outcome of life-threatening infections [30, 40, 41]. Collectively, these data support the hypothesis that measuring these markers at clinical presentation could facilitate triage, risk-stratification and precision management of malaria-infected patients.

#### Integrating severity markers into existing RDTs to identify cases of severe malaria

**Fig. 2:**
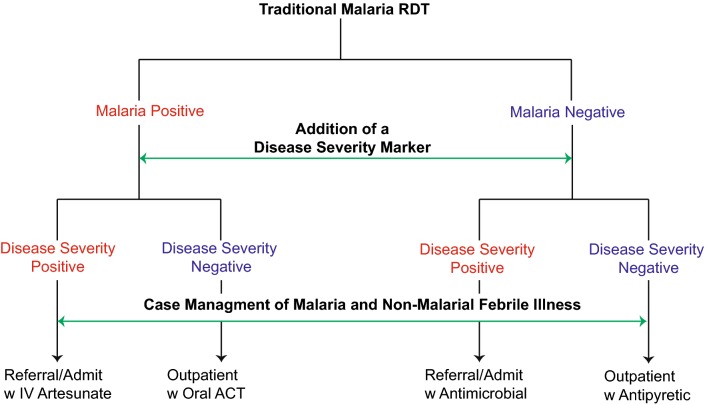
Proposed community-based management of febrile illness based on incorporating a disease severity marker into RDTs. An RDT with a disease severity marker could enhance triage and individualized management of children at risk of severe malaria or other life-threatening infections. In this pragmatic “precision medicine” approach, a febrile patient with a positive malaria result and negative disease severity result could managed as a case of uncomplicated malaria with an oral ACT treatment in the community setting. Patients with positive malaria results and positive disease severity results would be urgently referred for treatment with intravenous artesunate and supportive care. Patients with negative malaria but positive disease severity results would also be referred for supportive care and consideration of parenteral antibiotics. A patient with a malaria negative and disease severity negative result could be given antipyretics and monitored as an outpatient

RDTs are already widely implemented in community-based settings in Asia and Africa. These culturally acceptable and inexpensive diagnostic platforms could be adapted to incorporate a disease severity marker, enabling not only the detection of malaria, but simultaneously informing individualized management decisions regarding the need for referral and parenteral therapy (Fig. 2). In this proposed community-based approach, a febrile patient with a positive malaria result and negative disease severity result could be managed as uncomplicated malaria in the community with oral artemisinin-based combination therapy (ACT), whereas a patient with a positive malaria result and positive disease severity marker warrants urgent referral and parenteral artesunate.

#### Using markers of severity to risk-stratify non-malarial febrile illness

Depending on the location and season, up to 90% of RDTs used in the community setting in sub-Saharan Africa will be negative [8, 9, 14]. An integrated approach is needed to manage febrile illness and triage children at risk of life-threatening infection irrespective of aetiology. While additional prospective studies are needed, the triage approach described above for malaria, could also inform precision management for RDT-negative cases.

Most patients with non-malarial febrile illness have self-limiting infections [42]. Patients with impending severe infections require immediate referral for more advanced care and consideration for parenteral antimicrobial therapy [10, 43]. Enhanced pathogen-based diagnostics can potentially inform management with respect to antimicrobial therapy, but as in severe malaria, do not inform decisions as to which patients would most benefit from referral and hospital-based care.

For non-malarial fever, detailed studies have examined the utility of etiological data to guide triage and treatment [42]. However, acting on these data can be problematic due to the frequency of mixed infections—making assignment of causality challenging—further confounded by the high rate of carriage of pathogenic organisms in healthy controls. Rather than multiple pathogen-based approaches and the challenges posed by their logistics and interpretation by community health-workers, rapid tests for severe infections could have direct impact since in the absence of critical illness, most non-malarial febrile syndromes can be managed conservatively and without antimicrobials [43–46].

As above, markers of endothelial and immune activation (e.g. Angiopoietin/Tie2 axis, soluble triggering receptor expressed on myeloid cells-1 (sTREM-1)) predict clinical outcome in patients with non-malarial febrile illness, and could be used to risk-stratify patients and inform clinical management irrespective of aetiology [22, 26, 39, 47]. However additional studies are needed to further define their clinical utility, especially in low-resource community settings.

### Enabling triage of severe infections in community-based settings

Many deaths in children under five in low resource settings occur in remote regions. Rural place of residence is associated with an increased risk of delayed access to medical treatment and death before the age of five [48]. More than 50% of children in low resource settings die in the community without ever engaging the formal health care system [3, 48, 49]. Therefore, reducing under-five mortality will require triage tools that can be deployed in rural communities. This requires tools that are suitable for use by frontline community health workers and that empower them to make important management decisions at initial patient presentation. A “next generation” triage tool that incorporates a disease severity marker into existing RDTs would be appropriate for community-based triage and could improve case management of both malaria and non-malarial fever syndromes. Enhanced triage in the community could enable early detection of severe infections, facilitate timely referral and lead to improved health outcomes.

## Conclusions

The majority of febrile illness in children under the age of five is self-limited and, once malaria and critical illness are excluded, can be managed conservatively. The current inability to rapidly identify the small proportion of children who are at risk of progressing to life-threatening infection is a major obstacle to management of fever syndromes, rational antimicrobial use and effective health resource allocation. This paper proposes the incorporation of disease severity markers into existing RDTs as an approach to enable early recognition, risk stratification, and prompt treatment of severe malaria and other life-threatening infections. While additional studies are needed, this strategy could enhance triage, improve case management, resource allocation, and ultimately health outcomes for children presenting with both malarial and non-malarial febrile illness in hospital and community-based settings.

## Abbreviations

ACT: artemisinin-based combination therapy
CRP: C-reactive protein
RDT: rapid diagnostic test
PCT: procalcitonin
sTREM1: soluble triggering receptor expressed on myeloid cells-1
